# Soluble PD-L1 in Serum and Urine in Urinary Bladder Cancer Patients

**DOI:** 10.3390/cancers13225841

**Published:** 2021-11-21

**Authors:** Anders Vikerfors, Sabina Davidsson, Janusz Frey, Tomas Jerlström, Jessica Carlsson

**Affiliations:** Department of Urology, Faculty of Medicine and Health, Örebro University, 703 62 Örebro, Sweden; anders.vikerfors@regionorebrolan.se (A.V.); sabina.davidsson@regionorebrolan.se (S.D.); Janusz.Frey@regionorebrolan.se (J.F.); tomas.jerlstrom@regionorebrolan.se (T.J.)

**Keywords:** urinary bladder cancer, macroscopic hematuria, soluble PD-L1, serum, urine

## Abstract

**Simple Summary:**

Non-invasive diagnostic and prognostic biomarkers for urinary bladder cancer (BC) are eagerly awaited, since these could improve patient’s quality of life and reduce resources and costs for the health care system. The PD-1/PD-L1 axis is an important immune escape mechanism for tumors, and both high tissue expression and soluble levels of PD-L1 have been shown to be associated with worse prognosis for patients with various tumor. The knowledge about soluble PD-L1 in body fluids such as serum and urine in patients with BC is sparse, and more studies are needed to investigate its role as a biomarker for the disease. In the present study, we show that high serum PD-L1 levels are associated with aggressive disease and development of metastatic disease in BC patients.

**Abstract:**

Soluble PD-L1 (sPD-L1) levels have been identified as a potential biomarker for various cancers, but its diagnostic and prognostic value in urinary bladder cancer (BC) remains to be fully elucidated. In this study, we investigated sPD-L1 levels in serum and urine samples from 132 patients with BC and compared them to 51 patients with hematuria (controls). The levels of sPD-L1 in serum and urine were determined using ELISA. Soluble PD-L1 could be detected in 99.5% of the serum samples and 34.4% of the urine samples. Patients diagnosed with BC had significantly higher urinary levels of sPD-L1, compared to controls, however no difference were found in serum sPD-L1 levels (*p* = 0.038 and *p* = 0.61, respectively). Significantly higher serum sPD-L1 levels were found in patients with muscle invasive disease and metastatic disease, compared to patients with non-muscle invasive BC and non-metastatic disease (*p* < 0.05). There was also a trend for higher urine sPD-L1 levels in patients with metastatic disease, compared to patients with non-metastatic disease (*p* = 0.05). The results from this study suggest that sPD-L1 in serum, but not in urine, could be a potential prognostic biomarker for patients with BC.

## 1. Introduction

Urothelial bladder cancer (BC) is the ninth most common cancer worldwide with an estimation of 549,000 new diagnoses in 2018, and each year 200,000 people die due to the disease [1]. The majority of BC (~75%) initially present as superficial, non-muscle invasive (NMIBC) tumors that are confined to the urothelium or the lamina propria [2]. These tumors are characterized by a high recurrence rate, and progression to a muscle-invasive bladder cancer (MIBC) occurs in 10–30% among patients with high grade tumors [3]. Patients with a MIBC have a worse prognosis, with a 5-year survival rate of 20–35% compared to 60–80% in patients with NMIBC [4]. The high recurrence rate, treatment, and long-term follow-up make BC one of the most resource demanding and costly malignancies for the health care system [5]. Introduction of non-invasive diagnostic and prognostic biomarkers for BC are eagerly awaited since these could reduce the number of unnecessary cystoscopies, and improve both patient’s quality of life and reduce resources and costs for the health care system.

In order to survive and progress, tumor cells needs to evade destruction by the immune system. One mechanism for this is to deregulate immune checkpoints, which are involved in a plethora of pathways crucial for maintaining self-tolerance and for modulating the duration and amplitude of immune responses. One such immune checkpoint is the programmed cell death ligand 1 (PD-L1), which is a ligand to the co-inhibitory receptor programmed cell death 1 (PD-1). Binding of PD-L1 to PD-1 results in inhibition of the T-cell mediated immune response, which could result in tumor cells escaping immune surveillance [6,7]. The expression of PD-L1 in tumor tissue has been thoroughly investigated in patients with BC. High expression of PD-L1 has been associated with more advanced disease and worse prognosis compared to patients with a low PD-L1 expression [8,9,10,11]. However, there are contradictory results showing no association between PD-L1 expression and adverse clinicopathological features [12], as well as associations between high PD-L1 expression and good clinical outcome [13,14]. To the best of our knowledge, no study of PD-L1 expression and its relationship to diagnosis has been reported.

Both PD-1 and PD-L1 exist in soluble forms, namely soluble PD-1 (sPD-1) and soluble PD-L1 (sPD-L1) [15]. Soluble PD-L1 can be produced and released by both tumor cells and activated mature dendritic cells, while immature dendritic cells, macrophages, monocytes and T cells are refractory to releasing sPD-L1. The soluble form of PD-L1 is thought to be formed by proteolytic cleavage of an extracellular fraction of membrane bound PD-L1 (mPD-L1) [16]. Soluble PD-L1 is thought to retain its ability to bind membrane bound PD-1, and thus could inhibit T cell activation and proliferation [17]. Soluble PD-L1 has previously been detected in blood from patients with various cancers [18,19,20], and recently it was reported that sPD-L1 could also be detected in serum and urine samples in BC [21,22]. Further studies are however needed in order to investigate sPD-L1 in BC patients and our aims were to investigate if sPD-L1 levels in serum and urine could be a potential diagnostic and/or prognostic biomarker in BC.

## 2. Materials and Methods

### 2.1. Patients

Patients were recruited from the BLAdder cancer Blood and Urine Study (BLABUS), a prospective cohort study consisting of patients with macroscopic hematuria who were referred to the Department of Urology at the University hospital of Örebro for suspicion of bladder cancer. The vast majority of the patients underwent computed tomography urography, and an outpatient cystoscopy. Patients with visual suspicion of a bladder tumor at cystoscopy or urography thereafter underwent a transurethral resection of the bladder tumor (TUR-B) under general anesthesia. The diagnosis of BC was confirmed by a histopathological examination of the resected tissue samples. Tumor stage was defined according to the 2009 TNM classification of BC, and tumor grade was designated according to the WHO 2004 and WHO 1999 grading schemes. Patients without a history of BC and with a normal cystoscopy and urography were included as controls. Clinical data were extracted from medical records until 1 May 2019. Cause of death was defined as cancer specific if the patient had metastatic BC at time of death, and unrelated to BC if no signs of metastatic BC were detected at time of death. The study was conducted in accordance with the Declaration of Helsinki, and the protocol was approved by the ethics committee in Uppsala/Örebro region (approval number: 2016/088).

### 2.2. sPD-L1 Measurement

At inclusion, blood and urine samples were obtained from all patients, and were collected prior to any cystoscopy or treatment of the patient. Urine samples were centrifuged at 2000 rpm for 10 min, and the serum was obtained by centrifugation at 3000× *g* for 10 min at 4 °C. Both urine and serum samples were stored at −80 °C until analyses were performed. All samples were analyzed retrospectively, and samples from cases and controls were processed simultaneously. 

Soluble PD-L1 in serum and urine were measured by a commercially available ELISA for PD-L1, according to the manufacturer´s instructions (R&D Systems Inc., Minneapolis, MN, USA; Catalogue no. DB7H10). Control samples with known concentrations of PD-L1 (low, medium, and high) were included in each ELISA (R&D Systems Inc., Minneapolis, MN, USA; Catalogue no. QC226). The minimum detectable level of sPD-L1 in the ELISA was 4.52 pg/mL, and the detection range was 25.0–1600 pg/mL. The optical density was measured using a Multiskan Ascent plate reader (Thermo Fisher Scientific, Waltham, MA, USA) at 450 and 540 nm. 

Samples were measured in duplicates, and for each sample the coefficient of variation (CV, %) was calculated. The acceptable range of CV was 0–10%, samples with higher CV values were analyzed one more time. Blanks and standards were assayed according to the manufacturer´s instructions. The mean values of Absorbance vs. Concentrations were plotted and a 4-Parameter Logistic (4PL) non-linear regression model fit was applied, and R^2^-values above 0.9 were considered as acceptable. 

### 2.3. Statistics

In order to test the data for normality, a Shapiro-Wilks test was used. A Mann-Whitney U test or Kruskal-Wallis test were subsequently used to compare sPD-L1 levels between groups of patients. The urinary levels of sPD-L1 were dichotomized into absent or present, based on the detection level of sPD-L1 in the analysis method, and a Chi-square test was performed to test for association between dichotomized urinary sPD-L1 and cancer. Chi-square or Fisher´s exact tests were performed to test for associations between categorical variables. A Spearman correlation test was used to test for correlation between continuous data variables. Levels of sPD-L1 were subsequently divided into low or high, based on the median level of sPD-L1 among cases. The Kaplan-Meier method was used to estimate survival probabilities and the log-rank test was used to compare groups. A logistic regression model was used to estimate odds ratios (ORs) and 95% confidence intervals (CIs) of the association between sPD-L1 levels and metastatic disease. All analyses were performed in SPSS version 22.0 (IBM SPSS, New York, NY, USA). A statistical significance was considered at *p* < 0.05.

## 3. Results

A total of 132 BC patients and 51 controls were recruited between 1 October 2016 and 31 March 2019. Compared to controls, patients diagnosed with BC were significantly older at inclusion, they were more commonly current or former smokers, and of male sex. No difference in body mass index (BMI) at the time of inclusion were seen between cases and controls (Table 1). Of the 132 BC patients, 64 were included before undergoing TUR-B and the remaining 68 patients were included before undergoing a cystectomy. Twenty patients had primary metastatic disease or were diagnosed with metastatic disease during follow-up, and 13 patients died of BC during follow-up. The median follow-up time for BC patients were 7 months (range 0.8–29.7 months) (Table 2). For 14 patients, no evidence of a primary tumor were found during the histological examination of the urinary bladder after cystectomy, and were thus staged as T0. These patients were excluded from further analyses regarding sPD-L1 and its association with clinical parameters, leaving 118 cases left in the analyses.

### 3.1. sPD-L1 in Serum

Soluble PD-L1 could be detected in 182 of 183 (99.5%) of the available serum samples and the median concentration was 95.2 pg/mL (65.2–214.4 pg/mL). Men had significantly higher levels of sPD-L1, compared to women (*p* = 0.002). No correlation between sPD-L1 in serum and BMI, age, or smoking status were found. 

**Figure 1:**
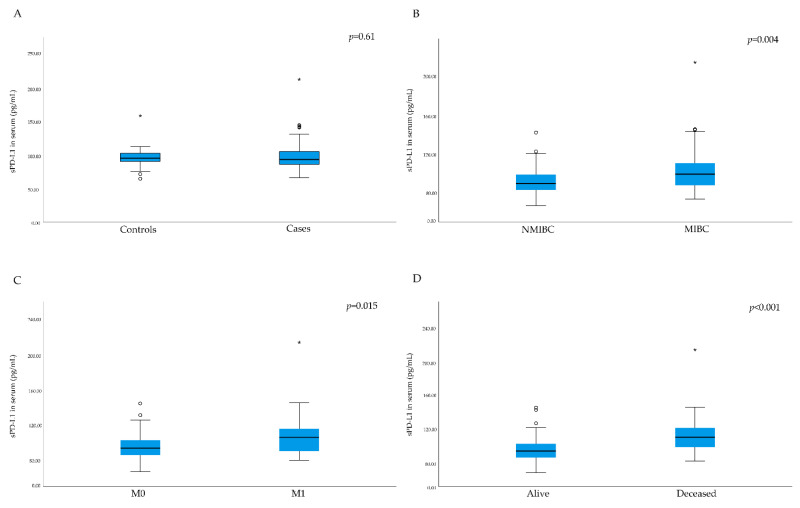
Box plots of serum levels of soluble programmed death ligand 1 (sPD-L1). (**A**) Across controls and cases. (**B**) Patients with non-muscular invasive disease (NMIBC) versus patients with muscular invasive disease (MIBC). (**C**) Patients with (M1) or without distant metastases (M0). (**D**) Patients who died during follow-up vs. patients who survived during the follow-up time. Outliers in the data are represented by a circle (°), and extreme outliers are represented by an asterisk (*).

No significant difference in serum levels of sPD-L1 between cases and controls were found (*p* = 0.61, Table 3); however, patients with MIBC had significantly higher sPD-L1 levels compared to patients with NMIBC (*p* = 0.004, Figure 1). Furthermore, patients with a high grade tumor had significantly higher levels of sPD-L1 compared to patients with a low grade tumor (*p* = 0.006). 

**Figure 2:**
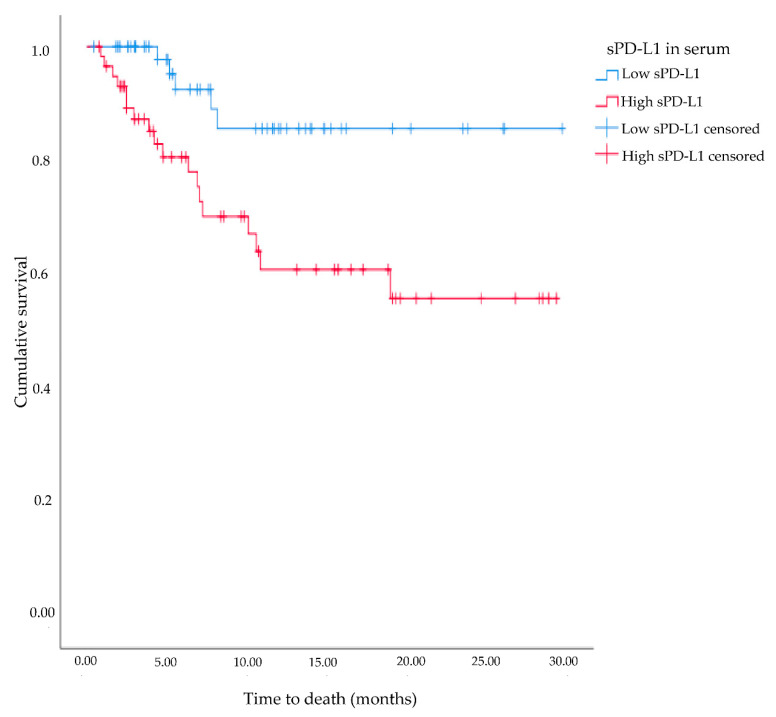
Kaplan-Meier survival plot for association of soluble programmed death ligand 1 (sPD-L1) levels (low vs. high) and overall survival among patients diagnosed with urinary bladder cancer between 2016 and 2019 within the BLAdder cancer Blood and Urine Study (BLABUS).

Significantly higher levels of serum sPD-L1 were found among patients who were diagnosed with metastatic disease or those who developed distant metastases during follow-up, compared to patients without metastatic disease (*p* = 0.015; Figure 1). A univariate logistic regression model showed that a one-unit increase in serum sPD-L1 increased the odds of having or developing distant metastases by 4.1% (*p* = 0.005, 95% CI: 1.01–1.07). When adjusting the model for tumor grade (low vs. high) and stage, this association was diminished, and no longer statistically significant (OR = 1.02, *p* = 0.24, 95% CI: 0.99–1.06). Patients that died during follow-up (all-cause mortality) had significantly higher serum sPD-L1 levels compared to patients who survived (*p* < 0.001; Figure 1), however no association with cancer-specific death was found (*p* = 0.29). A survival analysis showed that patients with sPD-L1 levels above median (94.0 pg/mL) had shorter overall survival time (19.6 months), compared to patients with sPD-L1 levels below the median value (26.4 months, *p* < 0.05, Figure 2).

### 3.2. sPD-L1 in Urine

Soluble PD-L1 could be detected in 56 of 163 (34.4%) of the available urine samples, and the median concentration was 74.2 pg/mL (57.5–669.2 pg/mL). No correlation between serum sPD-L1 and urinary sPD-L1 levels were found (R = 0.167, *p* = 0.22). Furthermore, no correlations were found between sPD-L1 in urine and BMI, age, or smoking status. 

Patients with BC had higher sPD-L1 levels in urine compared to controls (*p* = 0.038, Table 3), and sPD-L1 in urine tended to be more frequently detected in cases compared to controls (*p* = 0.07). No associations between sPD-L1 levels in urine and pT-stage and grade (low vs. high) were evident (*p* = 0.09 and *p* = 0.09, respectively), however patients who were diagnosed with metastatic disease or who developed distant metastases during follow-up had higher levels of urinary sPD-L1, compared to patients without metastatic disease (*p* = 0.05). No association between sPD-L1 levels in urine and all-cause mortality was found (*p* = 0.09).

## 4. Discussion

Urinary bladder cancer is a costly and resource demanding malignancy for the health care system, and introduction of non-invasive biomarkers are needed in order to reduce resources and costs for the health care systems. In this study, we measured the levels of sPD-L1 in serum and urine from 132 patients with BC and 51 controls with macroscopic hematuria and evaluated associations with clinicopathological characteristics. The results showed that sPD-L1 was detectable in the majority of the serum samples, but only in one third of the urine samples. Patients with BC had significantly higher urinary levels of sPD-L1, compared to patients without the disease; however, no difference between the groups were seen concerning serum levels. Furthermore, patients with a more aggressive form of BC, as indicated by muscle-invasive and/or metastatic disease, had higher serum levels of sPD-L1. These data are promising for further studies investigating the potential role of sPD-L1 as a prognostic biomarker for BC. 

Both pre-clinical and clinical studies have demonstrated that immune checkpoint blockade using anti-PD-L1 antibodies are successful as immunotherapy in several types of cancer, including BC [23,24,25,26]. Even though mPD-L1 is a commonly used biomarker for both prognosis and response to anti-PD-1/PD-L1 treatment in cancer, controversy remains over the predictive and prognostic utility of mPD-L1 positivity. The expression of mPD-L1 has been shown to have high intratumoral, intertumoral, and interpatient heterogeneity and the expression changes after treatments such as chemotherapy [27]. As the expression of mPD-L1 is not static, taking serial measurements could be required to monitor the disease and adjust treatments in a personalized manner. Compared to tumor tissue biopsies, blood and urine samples have the advantage of being easily accessible and could reflect different tumor clones present throughout the body. Finding circulating biomarkers in body fluids, that reflect disease state or treatment response, would therefore be of tremendous value for the clinic. Even though sPD-L1 is a promising biomarker for many cancers, one should keep in mind that circulating sPD-L1 only reflects mPD-L1 that has been shed of from tumor cells and mature dendritic cells, while T cells and macrophages are refractory to shedding of PD-L1. 

Increased serum levels of sPD-L1 in cancer patients, compared to healthy individuals, have been reported in e.g., renal cell cancer, hepatocellular cancer, and lung cancer [18,19,20]. In the present study, no significant difference in sPD-L1 levels in serum from patients with BC and controls were found. One of the reasons for this could be that the controls in this study were not healthy individuals, but instead patients referred to the Department of Urology for macroscopic hematuria. Even though hematuria is the most common symptom of BC, hematuria can also be caused by e.g., urinary tract infections, renal infections, or bladder stones. The main clinical aim in diagnosis of BC is to be able to separate between patients presenting with hematuria due to BC and patients presenting with hematuria due to other causes. Thus, when investigating diagnostic biomarkers for BC, having a control group of patients presenting with hematuria due to other causes than BC is a necessity. The results in the present study suggest that serum sPD-L1 levels cannot be used to separate between hematuria due to BC or other causes, and thus are not a suitable diagnostic biomarker for BC. However, studies in larger study populations are needed to investigate this further. 

Tosev et al., recently reported that patients with non-neoplastic diagnoses such as benign prostatic hyperplasia, chronic urocytitis, renal calculi, and one case of hematuria, had significantly lower urinary sPD-L1 levels compared to patients with BC [22]. This is in line with the results in our study. However, sPD-L1 were only detectable in one third of the urine samples in the present study, and no significant difference in the proportion of sPD-L1 positivity were found between cases and controls. Tosev et al., did not report the number of samples with undetectable sPD-L1 levels although the presented data showed that IQR includes 0, indicating undetectable levels among both controls and BC patients [22]. These results suggest that urinary sPD-L1 might be unsuitable as a diagnostic biomarker for BC, due to the low sensitivity. However, the reason to why only one third of the patients in the present study had detectable urinary sPD-L1 warrants further investigations. 

The results from the present study show that patients with MIBC had significantly higher serum sPD-L1 levels compared to patients with NMIBC, and that patients with metastatic disease had higher serum sPD-L1 levels compared to patients with non-metastatic disease. These results suggest that serum sPD-L1 levels increase with a more aggressive disease. Krafft et al., reported significantly higher levels of serum sPD-L1 among patients with high stage BC (T3–T4) compared to patients with low stage disease (T0–T2), although they found no association between sPD-L1 and lymphatic or visceral metastases [21]. Supporting data for increased levels of sPD-L1 in patients with metastatic disease has also been reported in other cancer types, such as non-small cell lung cancer and renal cell cancer [28,29]. High sPD-L1 levels were also associated with all-cause mortality in the present study, which is in line with data from Krafft et al., reporting sPD-L1 as an independent predictor of overall survival in patients with BC [21]. However, no association between sPD-L1 and cancer-specific death was found in the present study, possibly due to the small number of events, leaving the study without power for investigating this association. 

Even though there have been therapeutic breakthroughs for BC with the use of antibodies targeting PD-1 or PD-L1, there are still many questions that needs to be answered. One of the most important questions is why only a portion of the patients responds to immunotherapy. Thus, there is a demand for biomarkers that can identify responders versus non-responders prior to applying immunotherapy. Levels of sPD-L1 have previously been associated to treatment response to the immune checkpoint inhibitor Nivolumab (anti-PD-1) in patients with e.g., lung cancer [30]. Furthermore, Krafft et al., recently showed that high pre-treatment levels of sPD-L1 were associated with a poor prognosis for BC patients undergoing immunotherapy [21]. These studies indicate that sPD-L1 might also be a potential biomarker for patient selection to adjuvant immunotherapy.

A strength with the current study is the choice of controls, which reflects a clinical setting where the major aim is to separate between patients presenting with hematuria due to BC or other causes. We have also investigated the levels of sPD-L1 in both serum and urine samples from these patients. The study also has some limitations, mainly the small cohort size and the short follow-up time of the patients (median follow-up time; 7 months). Another limitation of this study is that the tissue expression of PD-L1 were not measured in these patients, thus making it impossible to compare mPD-L1 expression to sPD-L1 levels in serum and urine. Even though the study was underpowered for some of the analyses, the results suggest a potential value of serum sPD-L1 as a prognostic biomarker for BC, which motivates further investigations using larger study cohorts with longer follow-up time. 

## 5. Conclusions

Our results showed that sPD-L1 can be detected in both serum and urine samples in BC. Patients with BC had higher urinary sPD-L1 levels than controls, but since only a third of the samples had detectable levels it appears like urinary sPD-L1 is unsuitable as a biomarker for BC. High sPD-L1 levels, mainly in serum, were found to be associated with a more aggressive BC, as indicated by pT-stage and metastatic disease, and could thus be a potential prognostic biomarker for the disease. Further prospective and longitudinal studies are needed to assess serum sPD-L1 as a biomarker for monitoring of BC patients. 

## Figures and Tables

**Table 1:** Patient characteristics.

	All Patients	Cases	Controls	*p*
	*N* = 183	*N* = 132	*N* = 51	
**Age at inclusion** median (min-max)	73.4 (37.1–94.6)	74.2 (37.1–94.6)	67.6 (44.1–89.4)	<0.001 ^a^
**Sex**				0.006 ^b^
Male	128 (69.9)	100 (75.8)	28 (54.9)	
Female	55 (30.1)	32 (24.2)	23 (45.1)	
**Smoking**				0.003 ^b^
Yes	29 (15.8)	24 (18.2)	5 (9.8)	
No	90 (49.2)	55 (41.7)	35 (68.6)	
Former	6 (34.4)	53 (40.1)	10 (19.6)	
Missing	1 (0.5)	-	1 (2.0)	
**BMI** median (min-max)	25.6 (16.9–49.7)	25.7 (16.9–49.7)	25.2 (19.0–47.9)	0.96 ^a^

^a^ Mann-Whitney U test; ^b^ Chi-square test; BMI—Body mass index.

**Table 2:** Tumor characteristics of patients with urothelial bladder cancer (BC).

	BC Cases *N* = 132
**Point of inclusion**	
Pre-TURB	64 (48.5)
Pre-cystectomy	68 (51.5)
**Histology**	
Urothelial cancer	116 (87.9)
Squamous cell features	12 (9.1)
Sarcomatoid features	3 (2.3)
Missing	1 (0.8)
**Grade**	
1	13 (9.8)
2	25 (18.9)
3	72 (54.5)
Low grade	22 (16.7)
High grade	88 (66.7)
Missing	22 (16.7)
**cT-stage**	
Tis–T1	49 (37.1)
T2–4	71 (53.8)
Missing	12 (9.1)
**pT-stage**	
T0	14 (10.6)
Ta	27 (20.5)
Tis	5 (3.8)
T1	19 (14.4)
T2	30 (22.7)
T3	25 (18.9)
T4	12 (9.1)
**cN-stage**	
N0	125 (94.7)
N1	7 (5.3)
**pN-stage**	
N0	50 (37.9)
N1	30 (22.7)
Missing *	52 (39.4)
**M-stage**	
M0	104 (78.8)
M1	20 (15.2)
Missing	8 (6.1)
**Cause of Death**	
BC	13 (59.1)
Unrelated to BC	9 (40.9)
**Follow-up time** (months) Median (IQR)	7.0 (10.9)

Tx—no sign of tumor, T0 = No evidence of residual tumor after cystectomy; TUR-B—Transurethral resection of bladder, cT-stage—clinical T-stage, cN-stage- clinical N-stage, pT-stage—pathological T-stage, pN-stage—pathological N-stage, M-stage—Metastatic stage, BC—Urinary bladder cancer, IQR—Interquartile range; * No lymph node dissection performed.

**Table 3:** sPD-L1 concentration in serum and urine from patients with BC (cases) and patients with macroscopic hematuria without any evidence of BC (controls).

	sPD-L1 Serum			sPD-L1 Urine		
	Cases (pg/mL)	Controls (pg/mL)	*p*	Cases (pg/mL)	Controls (pg/mL)	*p*
N	132	50		45	11	
Median	94.1	96.1	0.61 ^a^	75.7	70.0	0.038 ^a^
IQR	19.0	12.5		19.0	10.7	
Min	66.9	65.2		60.6	57.5	
Max	214.4	159.6		669.2	142.0	

^a^ Mann-Whitney U test; IQR—Interquartile Range, sPD-L1—Soluble programmed death ligand 1, BC—Urinary bladder cancer, *p*—*p*-value.

## Data Availability

The data presented in this study are available on request from the corresponding author. The data are not publicly available due to ethical restrictions.
